# Evaluation of a short, all oral treatment regimen including bedaquiline, delamanid, linezolid, clofazimine, and pyrazinamide named “Regimen C” for pre-XDR tuberculosis in Niger

**DOI:** 10.3389/ftubr.2025.1718997

**Published:** 2026-01-13

**Authors:** Soumana Boubacar Soumana, Abdourahamane Yacouba, Cheikh Aboubacar Abdoul Lawi, Ibrahim Oumar, Bassirou Souleymane, Alphazazi Soumana, Tapha Ounoussa, Mahamadou Doutchi, Mamane Daou, Souleymane Brah, Eric Adehossi, Saidou Mamadou

**Affiliations:** 1 Université Abdou Moumouni, Faculté des Sciences de la Santé, Niamey, Niger; 2 National Reference Laboratory for Tuberculosis, Amirou Boubacar Diallo National Hospital, Niamey, Niger; 3 Ministry of Public Health and Hygiene, National Program Against Tuberculosis, Niamey, Niger; 4 Action Damien, Niamey, Niger; 5 Université André Salifou, Faculté des Sciences de la Santé, Zinder, Niger

**Keywords:** bedaquiline, delamanid, Niger, regimen C, XDR-tuberculosis

## Abstract

**Background:**

The emergence of extensively drug-resistant tuberculosis (XDR-TB) poses a serious challenge to global tuberculosis control, particularly in high-burden countries like Niger. In 2021, a new fully oral, shorter treatment regimen, named regimen C, was adopted nationally.

**Aim:**

This study aimed to assess its effectiveness under programmatic conditions.

**Methods:**

This was a retrospective, cross-sectional study conducted from April 2021 to December 2024. All patients with pre-XDR and XDR-TB treated in one of the four multidrug-resistant (MDR) TB units in Niger, who received the new standardized regimen and completed their treatment, were included in the study.

**Results:**

A total of 16 patients with pre-XDR-TB were included in the study. Clinical, microbiological, and radiological data were collected. The median age was 30.5 [interquartile range (IQR) 25–39 years], and 62.5% of patients were male. All patients had pulmonary pre-XDR TB. At the end of treatment, a therapeutic success rate of 75.0% was observed. Adverse events occurred in 88.0% of patients, including 2 cases (14.3%) of grade 4 adverse reactions. Undernourished patients tended to have an increased risk of unfavorable treatment outcomes, although this association was not statistically significant (*p* = 0.18).

**Conclusion:**

These findings suggest that the regimen evaluated in this study appears to be effective for the management of pre-XDR tuberculosis in Niger, with a promising treatment success rate.

## Introduction

Tuberculosis (TB) is an infectious disease that has existed for centuries, caused by *Mycobacterium tuberculosis* complex. It remains a major public health challenge globally and particularly in the West African sub-region, where living conditions increase the population's vulnerability (1). TB ranks among the leading causes of morbidity and mortality worldwide, affecting primarily populations in developing countries (1). It is often linked to precarious socio-economic conditions and underdeveloped health systems, making it a pressing public health concern—especially with the emergence of drug-resistant forms that hinder efforts to control the disease and make its treatment increasingly difficult (2).

Since the introduction of the first antibiotics, such as streptomycin in the 1960s, cases of resistance have been reported (3). Over the decades, the emergence of strains of *Mycobacterium tuberculosis* complex resistant to rifampicin, isoniazid (the two most powerful first-line anti-TB drugs, thus defining multidrug-resistant TB), and second-line drugs has become a growing concern in many regions of the world (4). Among these resistant forms, some are particularly difficult to treat, including pre-extensively drug-resistant (pre-XDR) tuberculosis. According to the World Health Organization (WHO), pre-XDR is defined as tuberculosis caused by *Mycobacterium tuberculosis* complex strains that are resistant to rifampicin, isoniazid, and fluoroquinolones. In contrast, XDR-TB is characterized by tuberculosis caused by *Mycobacterium tuberculosis* complex strains resistant to rifampicin, isoniazid, and additionally being resistant to any fluoroquinolone, as well as at least bedaquiline and or linezolide, all from Group A anti-TB drugs. Currently, the WHO classifies second-line anti-TB drugs into three groups: A, B, and C. Group A drugs are the most effective among second-line medications for treating drug-resistant tuberculosis and include levofloxacin, moxifloxacin, bedaquiline, and linezolid (5). XDR TB patients require individualized treatment regimens that utilize the most effective available drugs from Groups A to C, as determined by drug susceptibility test (DST) results and patient history. Older therapeutic protocols for pre-XDR TB involved over 18 months of treatment (6). These prolonged treatments required intensive monitoring due to their toxicity, in addition to being costly and often inaccessible in many countries (7, 8). All these factors made the implementation of such regimens particularly difficult. However, the availability of new and repurposed drugs [such as pretomanid (Pa), bedaquiline (B), and linezolid (L)] for managing drug-resistant TB, along with evidence from trials such as PRACTECAL (9), led the WHO to endorse a new, shorter, all-oral, standardized regimen (BPaL) for treating pre-XDR TB in patients who had not previously been exposed to these drugs (10). Over the past decade, Niger's drug-resistant TB program has adopted a cascade of regimen strategies, starting with the standard short treatment regimen (STR) based on second-line injectable drugs (SLIDs) and fluoroquinolones (FQs), with FQs serving as the core drug (11). Bedaquiline (Bdq), introduced in 2012, has been secured as the core drug for third-line regimens for patients who did not respond to or relapsed. This approach has achieved a definitive relapse-free cure rate of 84.7% (12). In 2016, the regimen became an adaptive short treatment regimen (aSTR), with a second-line injectable drug (SLID) and linezolid (Lzd).

The introduction of new and repurposed drugs for managing resistant TB prompted Niger drug-resistant committee in 2021 to develop a third-line regimen for treating pre-XDR and XDR-TB (13). This fully oral, short-course regimen, lasting 9 to 11 months, is referred to as “Regimen C.” Niger adopted this regimen in April 2021 for the management of pre-XDR and XDR-TB. Regimen C consists of a 4–6 months intensive phase (linezolid, high-dose isoniazid, bedaquiline, delamanid, clofazimine, and pyrazinamide), followed by a 5-month continuation phase (bedaquiline, delamanid, clofazimine, and pyrazinamide). The rationale of regimen C is that the bactericidal action of Lzd and high-dose isoniazid protects Bdq, while delamanid (Dlm) is included to protect the action of the other drugs. Clofazimine (Cfz) is used for its sterilizing properties, as is pyrazinamide, which also has synergistic activity with Bdq (14).

Four years after the implementation of this new regimen, it is timely to assess its effectiveness to help guide future public health interventions in the fight against drug-resistant TB in Niger—hence the importance of this study. This study aimed to evaluate the effectiveness of regimen C over 4 years. The goal is to provide real-world data on the performance of the protocol, thereby guiding clinical practice and public health policies in the fight against drug-resistant tuberculosis.

## Methodology

### Study setting

This study was conducted in the four MDR-TB units in Niger, located in Niamey, Maradi, Tahoua, and Zinder. The laboratory data were supplemented from the National Reference laboratory (NRL) for tuberculosis at the Amirou Boubacar Diallo National Hospital in Niamey, Niger.

### Study design and period

This was a cross-sectional study based on retrospective data collection over 4 years, from April 2021 to December 2024.

### Study population and sampling

The study's target population included all cases of drug-resistant tuberculosis in Niger. Data were collected using an exhaustive sampling approach across all MDR-TB treatment units. All patients diagnosed with pre-XDR or XDR tuberculosis received care in one of the country's four designated MDR-TB units.

### Inclusion and exclusion criteria

All patients with pre-XDR and XDR tuberculosis treated in one of the four MDR-TB units in Niger, who received regimen C and completed their treatment, were included in the study. Patients with pre-XDR or XDR-TB who were treated under the old regimen, as well as any patients with unusable records, were excluded.

### Data collection and analysis

Based on the criteria detailed above, data were collected using forms and review of medical records, along with information gathered from the electronic registries of patients from the four MDR-TB units. Collected data included sociodemographic data (age, sex, origin, occupation, marital status), medical history, comorbidities, and risk behaviors (smoking, alcoholism, etc.); clinical and radiographic data (body mass index, localization of tuberculosis, lung cavity), bacteriological data (sputum smear, sputum culture, Xpert MTB/RIF, MTB/XDR tests, test Hain) and therapeutic data (therapeutic status of patients, prior treatment with second line drugs, duration of treatment, side effects, and suspected causative drugs). Data were exported into the software programs Sphinx Plus version 5 and Epi Info 3.5 for analysis. A descriptive analysis was conducted, calculating frequencies and percentages for qualitative variables, as well as medians and interquartile ranges for quantitative variables. Percentages were compared using the chi-square test or Fisher's exact test. A *p*-value of less than 0.05 was considered statistically significant.

### Ethical considerations

This study was approved by the Faculty of Health Sciences of Abdou Moumouni University under the number 000033-2024/FSS/UAM/DOY/SP. Furthermore, approval for the study was obtained from the ethics committee of Niger (N°24/2021/CNERS). All collected information was anonymized and used solely for research purposes. Patient confidentiality was respected.

## Results

### Patient inclusion in the study

**Figure 1:**
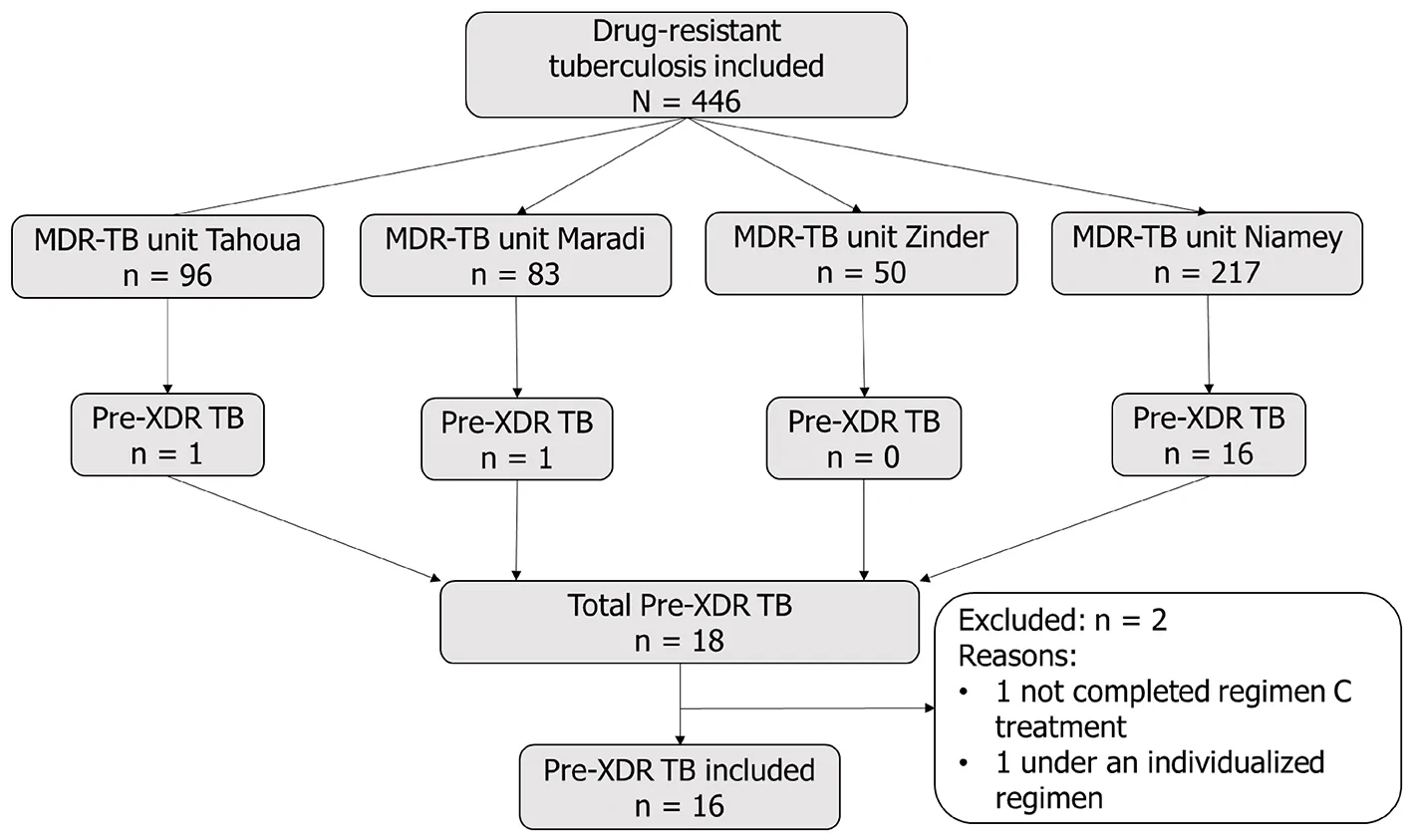
Flow diagram of inclusions.

A total of 446 cases of drug-resistant tuberculosis were identified from the four MDR-TB units in Niger. Of these, 18 cases of pre-XDR TB were identified. Two of the 18 cases of pre-XDR TB did not meet the inclusion criteria. Finally, 16 cases of pre-XDR TB under regimen C who completed the full treatment were included in the final analysis (Figure 1).

### Baseline characteristics of patients

The majority of patients were male (*n* = 10/16) with a sex ratio of 1.67. The median age was 30.5 years [interquartile range (IQR) 25–39 years], with age extremes ranging from 20 to 80 years. The age group ≤30 years was the most represented, accounting for 50.0% (*n* = 8/16).

Half (8/16) of patients with pre-XDR TB were undernourished (BMI <16.5 kg/m^2^). The average BMI was 16.07 ± 2.92 kg/m^2^. Among the 16 patients included, 6 were new cases, and 10 had previously been treated with either a drug-susceptible tuberculosis regimen or a regimen for rifampicin-resistant tuberculosis. None had received the earlier treatment regimen formerly used for pre-XDR TB. All patients were treated for pulmonary tuberculosis (Table 1).

**Table 1:** Basic characteristics of the study population.

**Characteristics**	**Number of cases, *n* (%)**
**Age groups (years)**	
≤30	8(50.0)
31–50	4(25.0)
≥51	4(25.0)
**Sex**	
Male	10(62.5)
Female	6(37.5)
**BMI groups (kg/m** ^2^ **)**	
<16.5	8(50.0)
16.5–18.49	6(37.5)
≥18.5	2(12.5)
Smoking history	3(18.7)
Prior treatment history	10(62.5)
Prior treatment with 2^nd^ line drugs	6(37.5)
**Therapeutic status of patients**	
New case	6(37.6)
Failure 1^st^ line*	5(31.2)
Failure 2^nd^ line**	1(6.2)
1^st^ line relapse	2(12.5)
Other***	2(12.5)
**Lung cavity**	
Unilateral	3(19.0)
Bilateral	13(81.0)

* Failure 1^st^ line: Patient previously treated with the regimen for drug-sensitive tuberculosis.

** Failure 2^nd^ line: Patient previously treated with the regimen for drug-resistant tuberculosis.

*** Other: Patient treated more than once for sensitive tuberculosis and resistant tuberculosis.

### Therapeutic data

All pre-XDR TB patients were treated with regimen C “4–6 LzdHhBdqDlmCfzZ/5 BdqDlmCfzZ,” except for one patient who received an individualized regimen C that consisted of “4 LzdHhBdqDlmCfzZ/2 HhImCsDlmBdqCfzZ/5 BdqDlmCfzZ.”

The treatment duration ranged from 9 to 11 months, with 12 out of 16 patients (75.0%) receiving a 9-month course.

During the treatment, 14 out of 16 patients (88.0%) experienced adverse drug reactions. Peripheral neuropathy and anemia were the most frequently observed, affecting 50.0% of patients, followed by hepatocellular injury in 35.7% (Table 2).

**Table 2:** Adverse effects experienced by patients.

**Safety outcomes**	**Number of cases, *n* (%)**
**Adverse effects**	
Peripheral neuropathies	7(50.0)
Anemia	7(50.0)
Elevated SGOT/SGPT	5(35.7)
Epigastralgia	3(21.4)
Kidney failure	3(21.4)
Qtc prolongation	1(7.1)
Diarrhea	1(7.1)
Visual disturbance	1(7.1)
**Grade of severity**	
Grade 1	6(42.9)
Grade 2	7(50.0)
Grade 3	6(42.9)
Grade 4	2(14.3)

### Bacteriological data

**Figure 2:**
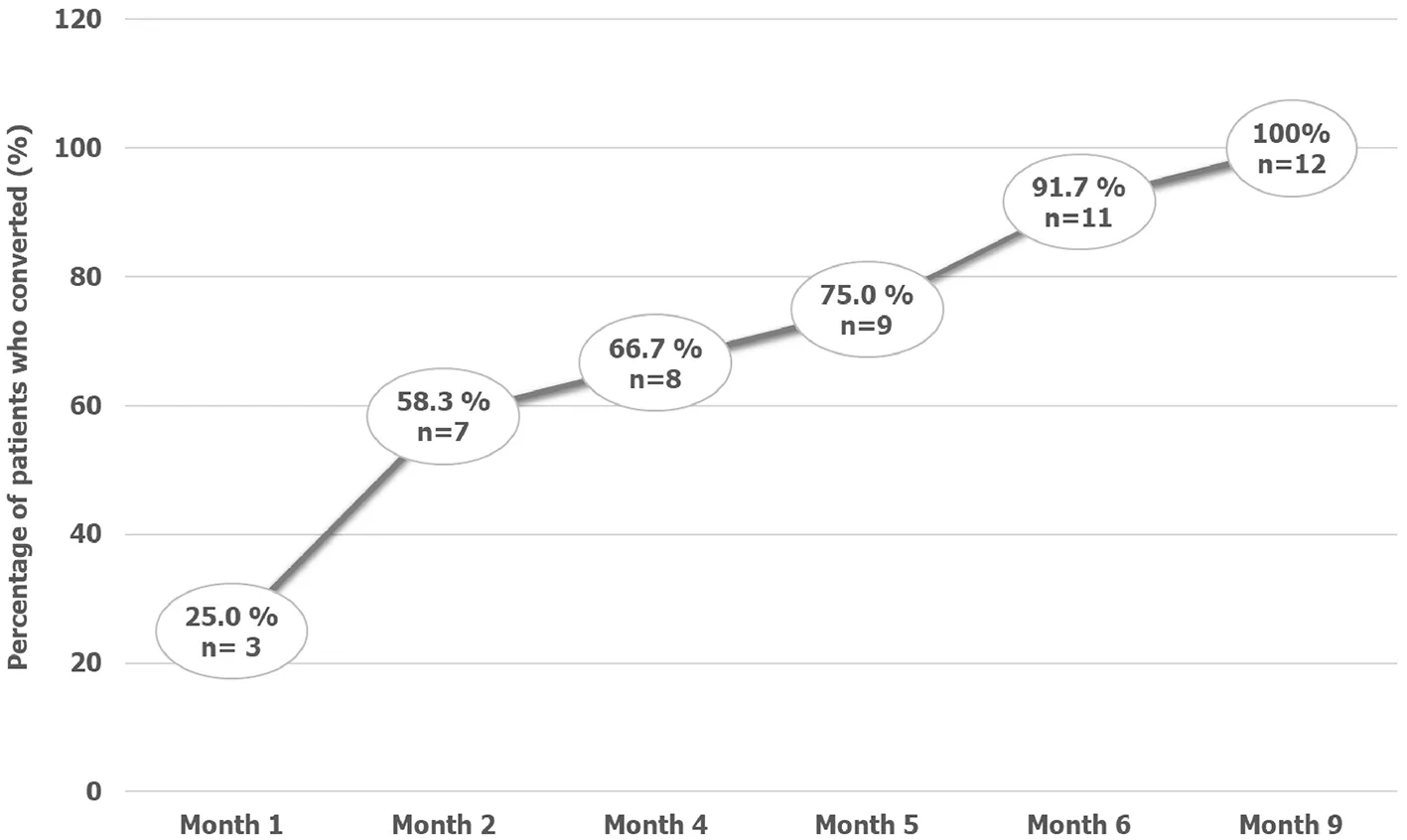
Sputum smear conversion month in patients who completed the treatment.

The frequency of sputum smear conversion increased progressively over time, reaching a peak of 91.7% by the end of the 6^th^ month among patients who completed treatment (Figure 2). As part of patient monitoring, 62.5% underwent culture testing at month 6, and all of them had negative cultures (Table 3).

**Table 3:** Bacteriological data.

**Bacteriological results**	**Number of cases, *n* (%)**
Resistance to fluoroquinolones	15(93.7)
“Mut 1” mutation	9(56.2)
Presence of *gyrA/gyrB* genes	4(25.0)
Presence of the *fabG* or *katG* genes	11(68.7)
Conversion of culture in the 4th month	13(81.2)
Conversion of culture in the 6th month	10(62.5)

According to the Hain Test, fluoroquinolone resistance was identified in 93.7% of patients (*n* = 15), and 68.7% (*n* = 11) were resistant to isoniazid, ethambutol, streptomycin, and fluoroquinolones. All patients showed mutations, with the “MUT1” mutation being the most common. Mutations conferring resistance to fluoroquinolones (*gyrA* and *gyrB*) were found in 4 patients (Table 3).

### Treatment outcomes

Of the 16 patients undergoing treatment, four unfavorable outcomes were reported. One patient dropped out after 1 month for personal reasons, while three patients died during treatment, with deaths occurring at 1 month, 2 months, and 5 months, respectively. Strikingly, three of the four unfavorable outcomes (75.0%) were observed in males who also presented with a BMI under 16.5 kg/m^2^. However, statistical analysis revealed no significant differences in age groups, BMI categories, history of tuberculosis, or adverse effects between the favorable and unfavorable groups (*p* > 0.05) (Table 4). Ultimately, 12 patients completed the treatment and were declared cured.

**Table 4:** Treatment outcomes.

**Variables**	**Items**	**Outcomes** ***n*** **(%)**		***p-value***
		**Favorable**	**Unfavorable**	
Sex	Male	7(58.3)	3(75.0)	0.99
	Female	5(41.7)	1(25.0)	
Body mass index	<16.5	5(41,7)	3(75.0)	0.18
	16.5–18.49	6(50.0)	0(0)	
	≥18.5	1(8.3)	1(25.0)	
History of tuberculosis	Yes	8(66.7)	2(50.0)	0.6
	No	4(33.3)	2(50.0)	
Adverse effects	Yes	11(91.7)	3(75.0)	0.45
	No	1(8.3)	1(25.0)	

## Discussion

Tuberculosis remains a public health problem in sub-Saharan Africa (15). In Niger, despite health policies implemented to combat it, tuberculosis continues to persist due to financial constraints, the population's standard of living, and insufficient health infrastructure. The emergence of pre-XDR and XDR-TB forms further hinders the global fight against this disease.

The significance of this study lies in its evaluation of the new standardized regimen for the management of pre-XDR and XDR-TB. These forms of drug-resistant TB are relatively rare, and no prior studies have been conducted on them in Niger.

The majority of pre-XDR patients were male, a finding that aligns with existing tuberculosis data from Niger and suggests that men have a higher exposure risk to tuberculosis than women (16). This trend may be attributed to several societal factors: men typically engage in more active professions that involve greater social interactions, while traditional norms—especially within the predominantly Muslim community—often lead to women participating less in public life (17). This result is consistent with reports from Ethiopia, where males made up 73.68% of pre-XDR-TB cases (18). However, Darrel et al. reported a higher proportion of females (51.1%) in the Congo in 2019 (19).

The most notable sociodemographic characteristic in this study is the age of the patients. The majority of pre-XDR-TB patients were young, with a median age of 30.5 years. This shows that drug-resistant TB affects both young and older individuals. Our results align with those of Li et al. in China, who found that 67.39% of their cohort were young people aged 15–44, with a median age of 38 (20). Similar results were also reported in Algeria and Zimbabwe (21, 22).

In this study, 87.5% of patients had a BMI below the normal threshold (18.5 kg/m^2^). Undernutrition is a common complication in tuberculosis (TB) patients, influenced by increased basal metabolism, chronic inflammation, and anorexia resulting from pro-inflammatory cytokines such as TNF-α, IL-1, and IL-6 (23). Additionally, some patients experience a loss of appetite due to common adverse effects associated with second-line anti-tuberculosis treatments (23). This sustained inflammation contributes to muscle protein breakdown and progressive weight loss (24–28). Additionally, socioeconomic factors such as food insecurity and poverty predispose some populations to malnutrition. Our results exceed those of previous reports in Nigeria, where 65% of patients had a BMI <18.5 kg/m^2^ (29).

In our study, 37.6% of patients were new TB cases, while 62.5% had a history of treatment. Of the latter, 30.0% relapsed, and the rest developed resistance during previous treatment (70.0%). Historically, Niger has implemented several treatment regimens for drug-resistant TB, beginning with the standard short treatment regimen (STR), which relied on second-line injectable drugs (SLIDs) and fluoroquinolones (FQs), with FQs serving as the core agents (11). Bedaquiline (Bdq), introduced in 2012, was subsequently established as the core drug for third-line regimens in patients who did not respond to or who relapsed after STR, contributing to a relapse-free cure rate of 84.7% (12). In 2016, the protocol transitioned to an adaptive short treatment regimen (aSTR), combining a SLID with linezolid (Lzd) (4–6 Am (Lzd)-Hh-Pto-Mfx-Cfz-E-Z/5 Mfx-Cfz-E-Z). Under this adaptive approach, SLIDs were replaced by Lzd.

The appearance of new pre-XDR-TB cases is concerning. In the absence of documented treatment history, these cases suggest primary transmission of resistant strains. Another aggravating factor in our context is the inappropriate and uncontrolled prescription of fluoroquinolones for common infections, creating selective pressure and promoting resistance mutations in *Mycobacterium tuberculosis* complex (30, 31). Similar findings were reported in Nigeria, with 62.2% of patients having a history of TB (32). Although this study did not find a statistical association between prior TB and treatment outcomes, literature consistently identifies TB history as a risk factor for recurrence (33, 34).

The regimen used in this study was regimen C, which consisted of a 4–6 months intensive phase (linezolid, high-dose isoniazid, bedaquiline, delamanid, clofazimine, and pyrazinamide), followed by a 5-month continuation phase (bedaquiline, delamanid, clofazimine, and pyrazinamide). One patient received a modified regimen with imipenem and cycloserine after stopping linezolid in month five. Treatment lasted 9 months for 75.0% of patients and 11 months for 18.7%. All patients had pulmonary pre-XDR TB. No resistance to WHO group A drugs was found, explaining the absence of XDR-TB cases in this study.

Smear conversion among patients completing treatment increased from 25.0% at 1 month to 58.3% at 2 months and 91.7% by 6 months. This demonstrates the importance of adherence to treatment for effective bacteriological conversion. Culture conversion at 6 months was 62.5%. Of the 6 patients without culture data, 3 had died, and 1 was lost to follow-up. These results are lower than Franke et al. (35), who found an 89% conversion rate, and Bastard et al., with 73% (36).

Anti-tuberculosis drugs are well known for their numerous adverse effects (37, 38). In our study, adverse drug reactions (ADRs) occurred in 88% of patients. The reported events included: peripheral neuropathy (grade 1 to 4), anemia (grade 2 and 3), elevated transaminases (grade 1 and 2), epigastric pain (grade 1), renal impairment (grade 1 and 2), diarrhea (grade 1), QT interval prolongation on ECG (grade 1), and visual disturbances (grade 1). These adverse effects are consistent with the known toxicity profile of the drugs administered (9, 39). Similar findings have been reported in other studies involving drugs included in our treatment regimen, notably bedaquiline, linezolid, clofazimine, and delamanid. For instance, the multicenter endTB study (16 countries) in 2022 reported the following adverse events: peripheral neuropathy (26.4%), QT prolongation (2.7%), acute renal failure (7.6%), and hepatotoxicity (5.5%) (40). These frequencies were lower than those observed in our cohort. In our study, peripheral neuropathy, anemia, and elevated transaminases were the most common adverse events, occurring in 50%, 50%, and 35.7% of patients, respectively. Peripheral neuropathy, commonly associated with linezolid and high-dose isoniazid, was managed with pyridoxine supplementation, NSAIDs, or amitriptyline, depending on severity. Anemia, frequently observed under linezolid and clofazimine, was treated with iron and folic acid supplementation or blood transfusions when necessary. QT prolongation, expected with bedaquiline and clofazimine, warranted close ECG monitoring. Hepatotoxicity, likely multifactorial but mainly attributable to pyrazinamide and isoniazid, requires rigorous laboratory monitoring.

This study reported a 75% treatment success rate, 6.3% loss to follow-up, and 18.7% mortality. Two-thirds of the deceased patients had prior TB treatment. Despite the non-negligible mortality, the standardized regimen C yielded promising outcomes. These results are comparable to the endTB-Q and BEAT India trials, which used similar drug combinations and achieved cure rates of 87% and 91%, respectively (41, 42).

Bivariate analysis showed a trend of higher adverse outcomes among patients with BMI <16.5 kg/m^2^, though not statistically significant (*p* = 0.18). In South Africa, Hosu et al. showed that underweight patients had 25.7% lower odds of treatment success than those with normal BMI, suggesting nutritional support should be a priority in managing resistant TB (43). This correlation between low BMI and poor treatment outcomes underscores the importance of integrating nutritional support into the management of drug-resistant tuberculosis.

Our findings suggest that males experienced more unfavorable outcomes (75%) than females (25%), though the association was not statistically significant (*p* = 0.999). A recent study performed in Cameroon found that males were 2.5 times more likely to have poor treatment outcomes (*p* = 0.006), consistent with our findings (44).

### Limitations

Several limitations of this study should be acknowledged. First, the main limitations of this study are its retrospective nature, the small number of cases, and the fact that the analysis focuses exclusively on pre-XDR tuberculosis cases as defined by the new WHO definition adopted in 2021, which limits the scope to this subpopulation and does not allow extrapolation to classic XDR-TB cases (45). The limited sample size significantly diminishes the statistical power required to generalize our findings to the wider population affected by pre–XDR tuberculosis. Second, while nutritional and clinical factors were suggested, they could not be explored in depth due to a lack of available data and structured nutritional monitoring. Third, no additional investigation was conducted into the patients' deaths to ascertain whether treatment was a direct cause. Some patients passed away at home, while others were reported deceased by their families weeks or even months later. Finally, drug resistance testing was not performed during the treatment follow-up. Nonetheless, this study offers valuable insights that can enhance surveillance of pre-XDR-TB in Niger and serves as foundational data for more extensive future research.

In perspective, this preliminary study lays the groundwork for more robust future research. Expanding the patient cohort, extending the study duration, and conducting investigations on a larger scale would help generate more relevant and generalizable data. Incorporating detailed clinical follow-up—including functional and physical assessments and nutritional parameters—as well as genomic diagnostic information would further strengthen the translational value of the findings. In addition, treatment monitoring should be more stringent and systematic, with updated drug-resistance testing, improved documentation of adverse events, and enhanced strategies for their management.

## Conclusion

Although conducted on a small cohort of pre-XDR-TB patients, this study has highlighted several important findings. The clinical and sociodemographic characteristics reveal concerning trends, notably the high proportion of undernourished patients, the young age of those affected, and the high frequency of adverse drug reactions.

The new regimen proves effective in the management of pre-XDR-TB in Niger, with an encouraging cure rate. Emphasis should be placed on strengthening nutritional support and improving monitoring of adverse effects. This preliminary study paves the way for larger, multicentric studies to confirm these hypotheses and bolster efforts against resistant tuberculosis in our country.

## Data Availability

The original contributions presented in the study are included in the article/supplementary material, further inquiries can be directed to the corresponding author.
